# Why Female Smokers Have Poorer Long-Term Health Outcomes than Male Smokers: The Role of Cigarette Smoking During Pregnancy

**DOI:** 10.3389/phrs.2024.1605579

**Published:** 2024-02-29

**Authors:** Li Yang, Yunchun Zhou, Mingyan Jiang, Wendy Wen, Yanfang Guo, Smita Pakhale, Shi Wu Wen

**Affiliations:** ^1^ Respiratory Medicine Department of Xiangtan Central Hospital of Hunan Province, Xiangtan, Hunan, China; ^2^ Clinical Epidemiology Program, Ottawa Hospital Research Institute, Ottawa, ON, Canada; ^3^ Department of Pulmonary and Critical Care Medicine of Yuxi People’s Hospital of Yunnan Province, Yuxi, Yunnan, China; ^4^ School of Epidemiology and Public Health, University of Ottawa Faculty of Medicine, Ottawa, ON, Canada; ^5^ BORN (Better Outcome Registry Network) Ontario, Children’s Hospital of Eastern Ontario, Ottawa, ON, Canada; ^6^ Division of Respiratory, The Ottawa Hospital, Ottawa, ON, Canada; ^7^ Department of Obstetrics and Gynecology, University of Ottawa, Ottawa, ON, Canada

**Keywords:** cigarette smoking, tobacco dependence, health effect, sex, female

## Abstract

**Objectives:** Women’s health status is better than men but the opposite is true for female smokers who usually have poorer long-health outcomes than male smokers. The objectives of this study were to thoroughly reviewed and analyzed relevant literature and to propose a hypothesis that may explain this paradox phenomenon.

**Methods:** We conducted a search of literature from three English databases (EMBASE, MEDLINE, and Google Scholar) from inception to 13 November 2023. A combination of key words and/or subject headings in English was applied, including relevant terms for cigarette smoking, sex/gender, pregnancy, and health indicators. We then performed analysis of the searched literature.

**Results:** Based on this review/analysis of literature, we proposed a hypothesis that may explain this paradox phenomenon: female smokers have worse long-term health outcomes than male smokers because some of them smoke during pregnancy, and the adverse effects of cigarette smoking during pregnancy is much stronger than cigarette smoking during non-pregnancy periods.

**Conclusion:** Approval of our pregnancy-amplification theory could provide additional evidence on the adverse effect on women’s long-term health outcomes for cigarette smoking during pregnancy.

## Introduction

Tobacco use is one of the most important risk factors for chronic diseases [1]. Globally, tobacco use accounted for about 7.69 million deaths and 200 million disability-adjusted life-years in 2019 alone [1]. On the other hand, tobacco use is a modifiable risk factor: quitting smoking will remove this risk. Tireless effort in the past decades has achieved some progresses in smoking cessation: prevalence of smoking had decreased among both males (27.5% reduction) and females (37.7% reduction) aged 15 years and older [1]. However, population growth has led to a significant increase in the total number of smokers: from 0.99 billion in 1990 to 1.14 billion in 2019 in the world [1]. Without continued effort in smoking cessation campaign, the annual toll of 7.69 million deaths and 200 million disability-adjusted life-years attributable to smoking will increase over the coming decades [1].

Health outcomes in female smokers are usually poorer than male smokers [2–4]. This is ironic as in general women have better health profile than men. According to the World Health Statistics 2018, women live longer than men in all regions, and the sex-gap in life expectancy was 4.3 years in 2000 and remained almost the same in 2016 (4.4 years) [5]. In this paper, we summarized studies comparing health outcomes between female smokers and male smokers, proposed study designs that could help explain this paradox phenomenon and discussed implications of the proposed studies.

## Methods

We conducted a search of literature from three English databases (EMBASE, MEDLINE, and Google Scholar) from inception to 13 November 2023. A combination of key words and/or subject headings in English was applied, including relevant terms for cigarette, tobacco, smoking, sex, gender, pregnancy, gestation, health, life expectancy, cardiovascular diseases, diabetes, and cancers. Articles that did not include health outcomes data for both sexes were excluded in the comparison of health status between males and females. Articles that did not include smoking and health outcomes for both sexes were excluded in the comparison of health outcomes between male smokers and female smokers. In addition, we searched for eligible studies through the reference lists of identified studies. Because of the broadness of the issues covered in this review, no attempt for a systematic review was made. As a result, the search is not considered exhaustive. Most of the included studies have addressed well-known facts such as the sex-gap in life-expectancy/general health status and impacts of pregnancy on women’s organ systems. For less certain topics such as sex-differences in smoking effects, we have tried to be neutral and have included reports for or against, if such a literature was identified in the search.

All the identified papers were analyzed by the research team and were organized into the following sections: sex-differences in general health status, differences in health effect between female smokers and male smokers, and impact of pregnancy on long-term health of cigarette smoking. For studies that were considered essential for analysis, namely differences in health effect between female smokers and male smokers, quality of the original studies was assessed using Newcastle-Ottawa Scale [6]. To help illustrate our points, we created hypothesized scenarios of the male-female differences in effects of smoking with cardiovascular disease risk (expressed as relative risks). In all these scenarios, we used never smoking as the reference and compared with different smoking status for males and females.

## Results

### Comparison of General Health Status Between Women and Men

It has been consistently demonstrated that women have superior health status than men. We summarized the recent worldwide statistics in Table 1 which showed that females have longer life-expectancy than males [5]. In 2000, worldwide life-expectancy in females was 4.3 years higher than males and in 2016 the difference was 4.4 years [5]. The female superiority in health has been observed in other health outcomes as well [7–16]. For example, studies found that female patients with chronic heart failure had better survival than male patients [11]. An analysis of 8,630 patients with first myocardial infarction event in Northern Sweden found a higher long-term survival rate in women than men [12]. A Taiwan population-based study showed that the 5-year overall and cancer-specific survivals of colorectal cancer were significantly higher in female patients than in male patients [14]. Chatkin et al. observed that in 253 patients with non-small cell lung cancer who had undergone lung resections, the 5-year survival rate for women (85.5%) was much higher than for men (46.6%) [16]. North and Christiani found that female lung cancer patients experienced higher survival than male patients, regardless of stage, histology, or treatment modality [16]. Overall, these observations suggest that regardless of diseases, clinical manifestations, and treatments, female patients in general experienced superior progresses and survival.

**TABLE 1:** Worldwide statistics on sex-specific life expectancy (Canada, 2023)^a^.

WHO region	Year	Life expectancy at birth (years)			Life expectancy at age 60 (years)		
		Both sexes	Male	Female	Both sexes	Male	Female
Global	2016	72.0	69.8	74.2	20.5	19.0	21.9
	2015	71.7	69.5	73.9	20.4	18.9	21.8
	2010	70.1	68.0	72.3	19.9	18.4	21.3
	2005	68.2	66.1	70.3	19.3	17.8	20.7
	2000	66.5	64.4	68.7	18.8	17.2	20.2
Africa	2016	61.2	59.6	62.7	16.6	15.9	17.3
	2015	60.7	59.1	62.2	16.6	15.8	17.3
	2010	57.6	56.4	58.8	16.1	15.4	16.7
	2005	53.4	52.3	54.4	15.4	14.7	16.1
	2000	50.8	49.6	52.1	15.0	14.3	15.6
Americas	2016	76.8	73.8	79.8	22.7	21.1	24.3
	2015	76.6	73.7	79.6	22.6	21.0	24.1
	2010	75.3	72.3	78.4	22.1	20.4	23.6
	2005	74.9	71.9	77.9	21.5	19.8	23.1
	2000	73.6	70.4	76.8	20.9	19.1	22.5
South-East Asia	2016	69.5	67.9	71.3	18.2	17.2	19.14
	2015	69.2	67.6	70.9	18.1	17.1	19.0
	2010	67.4	66.1	68.7	17.6	16.7	18.4
	2005	65.4	64.4	66.4	17.0	16.2	17.8
	2000	63.5	62.5	64.4	16.7	15.8	17.5
Europe	2016	77.5	74.2	80.8	22.3	20.2	24.1
	2015	77.2	73.8	80.5	22.1	20.0	23.9
	2010	75.7	72.0	79.3	21.4	19.2	23.2
	2005	73.5	69.5	77.6	20.3	18.1	22.3
	2000	72.5	68.4	76.7	19.7	17.3	21.6
Eastern Mediterranean	2016	69.1	67.7	70.7	18.2	17.5	19.0
	2015	68.8	67.4	70.4	18.1	17.4	18.8
	2010	68.1	66.7	69.5	18.0	17.3	18.7
	2005	66.2	64.8	67.8	17.6	16.9	18.4
	2000	65.5	64.2	66.9	17.5	16.8	18.2
Western Pacific	2016	76.9	75.0	78.9	21.0	19.5	22.5
	2015	76.7	74.8	78.8	20.9	19.4	22.4
	2010	75.8	73.8	77.9	20.5	19.0	22.1
	2005	74.7	72.7	76.8	20.1	18.6	21.6
	2000	72.8	70.8	75.0	19.3	17.8	20.9

^a^ Source: From World Health Organization Data. Available at: http://apps.who.int/gho/data/view.main.SDG2016LEXREGv?lang=en. (Accessed 14 January 2021).

### Comparison of Health Outcomes Between Female Smokers and Male Smokers

Contrary to the situation in the general population, female smokers were usually at greater risk for smoking-related chronic diseases than male smokers [2–4, 17–40]. We summarized recent studies that provided numerical comparison on the effects of cigarette smoking on chronic diseases between women and men (Table 2), which showed that in general, female smokers were at greater risk for smoking-related chronic diseases than male smokers. All the included studies were cohort or case-controls studies, with fairly good quality (Table 3). Powell et al observed that moderate/heavy female smokers were at higher risk for lung cancer than male smokers with the same amount of smoking [4]. This sex-difference in effects of cigarette smoking was particularly prominent for squamous and small cell lung cancers [31–34]. Female smokers had an increased risk for advanced colorectal neoplasia than male smokers [35]. In a longitudinal population study in UK, Prescott et al found that females who smoked cigarette had approximately a 50% increased risk of dying from vascular diseases than males who smoked cigarette [2]. In a systematic review, Mongraw-Chaffin et al summarized studies comparing the effect of cigarette smoking on coronary heart disease between males and females and found that in average female smokers had a 25% greater risk of coronary heart disease than male smokers [36]. Jamee et al made the same observation in a study in Gaza-Palestine [37]. Female smokers were more likely to experience cardiovascular complications than male smokers [38]. Another meta-analysis of the association between cigarette smoking and stroke found that current smokers had an increased risk of stroke compared with non-smokers (Odds ration (OR): 1.46, 95% CI: 1.04–2.07, *p* < .023), which was influenced by sex (OR in men 1.54 and OR in women 1.88) [21]. Female smokers with chronic obstructive pulmonary disease (COPD) were easier to develop airway obstruction after fewer numbers of cigarettes per lifetime than male smokers, and women with severe COPD have a 50% increased risk of death compared to men [39].

**TABLE 2:** Sex-specific effect of smoking on chronic diseases (Canada, 2023).

Author	Publication year	Diseases	Sample size	Smoking quantity	Male	Female
					*Odds ratio* (95% *Confidential interval*)	*Odds ratio* (95% *Confidential interval*)
Ross [21]	1992	Lung cancer	14,596	40≤ Pack-year <50	11.6 (7.7–17.6)	21.4 (14.3–32.2)
				Pack-year ≥50	13.8 (9.2–20.9)	32.7 (19.0–56.2)
Kreuzer [22]	2000	Lung cancer	4,623	30+Cig/day	2.4 (2.0–2.8)	3.4 (1.6–7.3)
Powell [4]	2013	Lung cancer	60,042	Moderate	8.2 (7.4–9.2)	10.8 (9.6–12.1)
				Heavy/very heavy	12.8 (11.5–14.2)	19.1 (17.0–21.5)
McGee [23]	2004	Coronary heart disease	344,671	Not mentioned	0.6 (0.5–0.7)	0.6 (0.5–0.7)
Asia Pacific cohort studies collaboration [24]	2005	Coronary heart disease	3,976	The lower mean daily cigarette consumption	1.6 (1.4–1.7)	1.7 (1.5–2.0)
Majken [25]	2008	Coronary heart disease	685	Light (1–14 g/d)	2.5 (1.8–3.4)	1.6 (1.2–2.0)
			951	Heavy (>15 g/d)	3.3 (2.5–4.4)	2.4 (2.1–2.8)
Jousilahti [26]	1999	Coronary heart disease	14,783	Not available	1.8 (1.5–2.1)	2.1 (1.5–3.1)
Nilsson [27]	2006	Coronary heart disease	32,715	Not available	2.3 (2.1–2.5)	3.2 (2.5–4.0)
Schnohr [28]	2002	Coronary heart disease	Not available	Not available	1.4 (1.2–1.6)	2.0 (1.8–2.3)
Inger Njølstad [16]	1996	Coronary heart disease	11,843	Not available	1.9 (1.6–2.3)	3.3 (2.1–5.1)
Auni [17]	2004	Coronary heart disease	1,296	Not available	3.3 (1.7–6.5)	8.8 (1.5–51.8)
Fraser [18]	1992	Coronary heart disease	2,254	Not available	0.9 (0.1–6.9)	2.4 (0.3–22.7)
Pan [20]	2019	Stoke	30,3134	Not available	1.54 (1.11–2.13)	1.88 (1.45–2.44)

**TABLE 3:** Quality assessment of the included studies in Table 2 by Newcastle-Ottawa Scale for assessing the quality of the included studies (Canada, 2023)^a^.

First author, year	Study design	Selection	Comparability	Exposure/outcome	Total scores
Powell, 2013 [4]	Retrospective cohort study	★★★	★★	★★★★	9
Inger Njølstadl, 1996 [16]	Prospective study	★★★	★★	★★★	8
Aunil, 2004 [17]	Prospective cohort study	★★★★	★★	★★★	9
Fraserl, 1992 [18]	Retrospective cohort study	★★★	★★	★★	7
Panl, 2019 [20]	Retrospective cohort study	★★★	★★	★★★	**8**
Rossl, 1992 [21]	Case-cohort & case-control	★★★	★★	★★★	8
Kreuzerl, 2000 [22]	Multicentre case-control study	★★★	★★	★★★	8
McGeel, 2004 [23]	Retrospective cohort study	★★★	★★	★★	7
Asia Pacific Cohort Studies Collaboration l, 2005 [24]	Retrospective cohort study	★★★★	★★	★★★	9
Majkenl, 2008 [25]	Prospective cohort study	★★★★	★★	★★★	9
Jousilahtil, 1999 [26]	Prospective cohort Study	★★★	★★	★★★	8
Nilssonl, 2006 [27]	Case-cohort & case-control	★★★	★★	★★★	8
Schnohrl, 2002 [28]	Prospective cohort study	★★★★	★★	★★★	9

^a^ The Newcastle-Ottawa Scale quality instrument is scored by awarding a point for each answer that is marked with an asterisk; Possible total points are 4 points for Selection, 2 points for Comparability, and 3 points for Outcomes.

Different from its harmful effect on most chronic diseases, cigarette smoking had a protective effect on Parkinson’s disease [40]. A meta-analysis of the association between cigarette smoking and Parkinson’s disease found that the protective effect of smoking against Parkinson’s disease was greater in male smokers than in female smokers [40]. We do not know the mechanisms of the protective effect of cigarette smoking on Parkinson’s disease. Regardless of the mechanisms, however, the results from a single smoking-disease association could not alter the hypothesis we have proposed in this paper that was based on majority of the relevant studies that we could identify.

### Physiological Changes During Pregnancy and Its Potential Role on Long-Term Health Effects Among Female Smokers

Most women will go through pregnancy at some point in their life, and the female body undergoes tremendous anatomical and physiological changes, such as changes in cardiovascular, respiratory, endocrine, haematologic, renal, and gastrointestinal systems to accommodate the metabolic and physical demands of pregnancy [41–60]. Blood volume in pregnant women increases 30%–50% [41, 44]. Heart-beat rate increases by 15–20 beats per minute [40, 45] and cardiac output increases up to 30%–50% [41, 45] during pregnancy. The pH and PaO2 in the arterial blood gas during pregnancy are higher than nonpregnancy period while the PaCO2 are lower in pregnancy [52]. Alternations in hormone levels during pregnancy could induce postpartum anxiety [53–57]. The expanding of uterus during pregnancy could displace the digestive organs [41]. β-cells could be dilated in order to meet the metabolic demands of pregnancy [59]. Pregnancy could also trigger some diseases such as asthma attack [59].

Although many of the changes during pregnancy will gradually return to normal levels after childbirth, some changes, especially pathophysiological ones, are likely to be lifelong [61–63]. These changes could lead to increased absorption and circulation of the toxic substances contained in cigarette smoking, and therefore amplify the harm of cigarette smoking for female smokers, increasing the risks of both short-term pregnancy complications and long-term health outcomes. Previous studies have found associations of pregnancy complications with long-term health outcomes. In a study that followed 12,849 women affected by preeclampsia and 284,188 women without, van Walraven et al found that venous thromboembolism was more common in the preeclampsia group (0.12%, 41.7 events per 100 000 person years observation) than in any of the control groups (range 0.01%–0.08%, rates of 3.0–33.8 events per 100,000 person years observation) [61]. Based on a follow up study data, Smith et al estimated that a total of 18.2% of preeclamptic women and 1.7% of control women had a high 10-year risk, 31.3% of preeclamptic women and 5.1% of control women had a high 30-year risk, and 41.4% of preeclamptic women and 17.8% of control women had a high lifetime risk for cardiovascular disease [62]. In a longitudinal follow up of a large group of pregnant women (including both with or without gestational diabetes), Retnakaran & Shah found that each 1 mmol/L increment in the glucose challenge test result was associated with a 13% higher risk of cardiovascular disease after adjustment for age, ethnicity, income, and rurality [63]. Although direct evidence from human study on how pregnancy amplification effects for cigarette smoking is not available, animal disease models on metabolisms in pregnancy [64, 65] suggest such a possibility.

### How to Explain the Differences of Health Effects Between Male Smokers and Female Smokers?

Cigarette smoking is causally responsible for many chronic diseases [66]. Around 140,000 premature deaths from cardiovascular diseases were caused by smoking in the United States each year [67]. Some researchers observed that ischemic heart disease incidence rises with increased dose of cigarette smoking [67, 68]. Nicotine could cross the placenta and concentrate in fetal blood and amniotic fluid in pregnant women who smoke cigarettes during pregnancy [69].

Smoking during pregnancy is an important risk factor for adverse outcomes for pregnant mothers and neonates [70–82]. Maternal smoking has also been linked to attention-deficit hyperactivity disorder, learning disabilities, behavioral problems, and increased the risk of nicotine addiction in the offspring [83–87]. Long term effects of maternal smoking on the offspring include obesity, type 2 diabetes, hypertension, asthma, COPD, childhood cancers, and reduced fertility in the offspring [87–92]. However, there are limited studies on the association between smoking during pregnancy and the long-term outcomes in women themselves.

**FIGURE 1:**
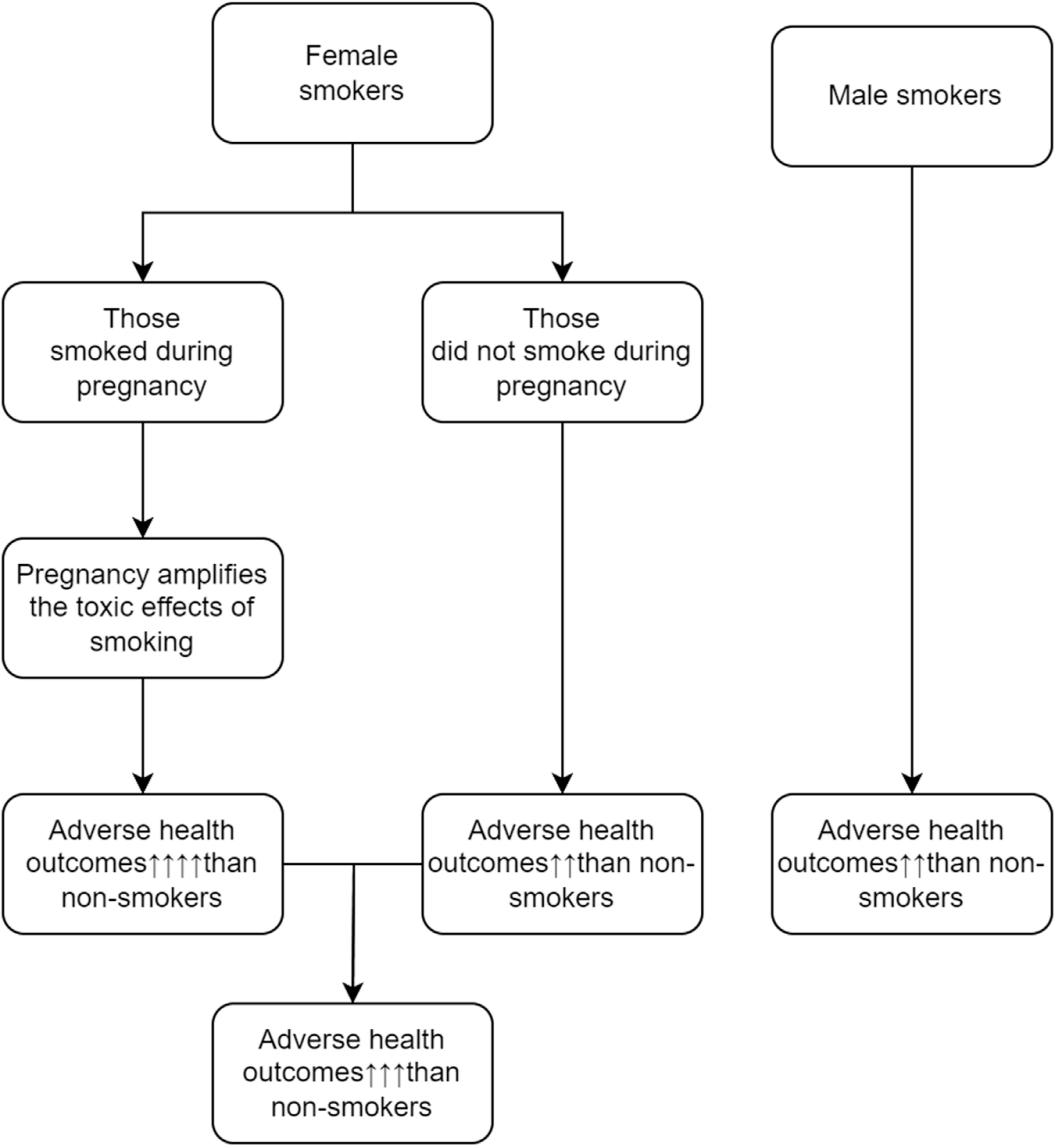
Potential mechanisms that may explain why female smokers have poorer long-term health outcomes (Canada, 2023).

Reasons behind sex differences in the effects of cigarette smoking are not clearly understood. Some investigators suggested that male-female differences in genetic susceptibility, hormones, and exposure to other environmental factors may explain the male-female differences in health hazards of cigarette smoking. We hypothesize that female smokers have worse long-term health outcomes than male smokers because some of them smoke during pregnancy, and the adverse effect of cigarette smoking during pregnancy is much stronger than the effect of cigarette smoking during non-pregnancy period. The graphic presentation of the potential mechanisms that may explain why female smokers have poorer long-term health outcomes as compared with male smokers is displayed in Figure 1.

Although an association between maternal cigarette smoking and decreased risk of preeclampsia and gestational hypertension has been observed [93, 94], this did not provide evidence against our proposal. Preeclampsia/gestational hypertension is a heterogeneous entity, with severe preeclampsia/gestational hypertension carries short- and long-term consequences to the mother and their children, mild preeclampsia/gestational hypertension does not seem affect the health of the mother and their children [94]. Maternal cigarette smoking, while reduce the incidence of preeclampsia/gestational hypertension, mortality and morbidity in smokers who developed preeclampsia/gestational hypertension during pregnancy were higher in non-smokers who developed preeclampsia/gestational hypertension during pregnancy [95–97]. This observation suggests that cigarette smoking may reduce the incidence of mild form of preeclampsia/gestational hypertension but may increase the severity of preeclampsia/gestational hypertension [95–97]. Because long-term health outcomes are more likely associated with severe form of preeclampsia/gestational hypertension [62], maternal cigarette smoking carries long-term harm to them.

Hypotheses other than pregnancy on the higher risk of cigarette smoking in female smokers than in male smokers, including differences in genetics, hormones, environmental exposures (including passive smoking), and difficulties in quitting smoking have been proposed [98–103]. Possible interaction between smoking and hormonal factors may need to be considered in the observed male-female difference in smoking effects. Elevated arginine vasopressin level has been associated with chronic diseases including cardiovascular diseases [99]. In a small study, Guaderrama at al observed that female smokers had higher arginine vasopressin level than male smokers [99]. In a study in women undergoing *in-vitro* fertilization treatment, active smokers (defined as smoking at least one cigarette per day at the time of procedure) had significantly lower anti-mullerian hormone levels and antral follicle counts, and worse overall *in-vitro* fertilization outcomes than non-smokers (defined as never smoke or who quitted smoke for 1 year prior to the treatment) [100, 101]. Bennett et al [102] reported that for never-smoking women, passive smoking was responsible for between 42% and 49% of the lung cancer cases. However, this type of study could not help to explain the difference of health outcomes in male smokers versus female smokers. In a meta-analysis of the 14 placebo-controlled nicotine patch trials (N = 6,250) for which long-term (6 months) clinical outcome results could be determined separately by sex, women had greater difficulty quitting smoking [103]. The reasons why female smokers are more difficult to quit smoking remains unknown, however.

### Previous Studies Comparing Female and Male Differences in Health Effect of Cigarette Smoking

Previous studies comparing differences in the effects of cigarette smoking between female smokers and male smokers are limited by the lack of an appropriate control group to test a hypothesis. A direct comparison between male smokers with female smokers can reveal the sex difference in health effect of cigarette smoking. However, there are major differences in anatomy, physiology, genetics, and exposure to varies environmental factors which may confound the association of smoking with sex. All these differences could explain the smoking effects, but could not test a hypothesis why female smokers have poorer long-term health outcomes on specific and unique female factors.

The sex differences in anatomy, physiology, genetics, and exposure to environmental factors, on the other hand, are usually not modifiable, so a study solely comparing outcomes between male and female smokers may have limited implication in terms of public health and disease prevention. A comparison of long-term health outcomes between female smokers who are childbearing and who are childless may be interesting. However, very rare for a study examined the rate of cigarette smoking and its association with long-term outcomes in childless women specifically. We made specific search of literature on this topic and could not identify a published study that compared long-term health outcomes between female smokers who are childbearing and those who are childless.

### Proposed Studies to Test the Hypothesis of Pregnancy Amplifies the Long-Term Harm of Cigarette Smoking in Female Smokers

Our hypothesis that pregnancy amplifies the consequence of cigarette smoking in female smokers, if proven true, has major implication in prevention, as it emphasizes the importance of smoking cessation during pregnancy in terms of both short- and long-term health outcomes.

It is rather difficult to test the hypothesis that effect of tobacco smoking during pregnancy is stronger than tobacco smoking during non-pregnancy periods directly, because of the difficulties to separate the person-time from the same woman during pregnancy period versus non-pregnancy period and relate that to outcome. As a result, we propose a simple approach by comparing the effects of female smokers versus male smokers: overall female smokers, restricting to female smokers who quitted smoking during pregnancy, and restricting to female smokers who were smoking free during pregnancy and thereafter. We created hypothetical scenarios to illustrate our points, using cardiovascular disease as an example (Table 4). In this example, under the ideal scenario (female smokers who quitted smoking in pregnancy entirely and maintained smoke free postpartum entirely), the effect of tobacco smoking in female smokers on cardiovascular disease (1.25) should be smaller than male smokers (1.75), consisting with the better general health status in females than in males.

**TABLE 4:** Effects of tobacco smoking on cardiovascular disease between female smokers and male smokers under different hypothetic scenarios (Canada, 2023)^a^.

Scenario	Relative risk for females	Relative risk for males
All female smokers included	2.50	1.75
Excluding female smokers who quitted smoking in pregnancy entirely^b^	1.75	1.75
Excluding female smokers who quitted smoking in pregnancy partially^c^	2.00	1.75
Excluding female smokers who quitted smoking in pregnancy entirely and maintained smoke free postpartum entirely	1.25	1.75
Excluding female smokers who quitted smoking during pregnancy entirely and maintained smoke free postpartum partially	1.50	1.75
Excluding female smokers who quitted smoking during pregnancy partially and maintained smoke free postpartum entirely	1.75	1.75
Excluding female smokers who quitted smoking during pregnancy partially and maintained smoke free postpartum partially	2.25	1.75

^a^ Never smoking as the reference in all analysis.

^b^ Defined as “no smoke” for all encounters in pregnancy (or postpartum) periods.

^c^ Defined as “no smoke” for some of the encounters in pregnancy (or postpartum) periods.

Under the ideal scenario (female smokers who quitted smoking in pregnancy entirely and maintained smoke free postpartum entirely the effect of tobacco), the effect of tobacco smoking in female smokers on cardiovascular disease (1.25) should be smaller than male smokers (1.75).

Another method to test this hypothesis is to compare long-term health outcomes between female smokers who had children and those who were childless. If the outcomes are poorer for female smokers who were childbearing as compare with female smokers who were childless, our hypothesis should be valid as for women who were childless all smoking were non-pregnancy while for women who were childbearing at least part of smoking were pregnancy.

## Discussion

Changing human behaviours is one of the most difficult tasks for public health effort. Despite many studies showings the harmful effects of cigarette smoking, about 10%–17% of women smoked during pregnancy worldwide [104–109]. Of those smoked during pregnancy, 40% quitted at the first trimester of pregnancy. However, about 60% of those who quitted smoking in pregnancy resumed smoking 6 months postpartum [108, 109]. These statistics indicate the challenges we are facing and that more evidence from solid studies is needed, including the ones proposed in this paper.

Limitations of our analysis should be acknowledged. First, because of the scope and breadth of the issues addressed in this paper, we have not attempted to conduct a systematic review. The lack of systematic review may result in biased assessment of the adverse health effect between female smokers and male smokers and between pregnancy smoking and non-pregnancy smoking. Systematic review is still needed to resolve this paradox phenomenon in health effects of smoking. Second, because of the limited number of identified relevant original studies and because of major heterogeneity of the included studies, we have not been able to summarize the evidence by a meta-analysis. The inability to make a meta-analysis for the included original studies made it necessary in some degree of arbitrary in analysis and cautions should apply in generalizing the conclusions based on this type of analysis. Third, no data from childless women was available, so we could not make a direct comparison of the health effect between smoking during pregnancy versus smoking in no pregnant women. Fourth, because of the unavailability in original studies, in the comparison of general health status between males and females, smokers were not excluded. Fifth, the theory that pregnancy amplifies the harmful effects of cigarette smoking was inferred indirectly from physiological changes during pregnancy and from animal experiments, not from direct human studies. Finally, sex differences in socio-economic status, access to healthcare, and rates of smoking, alcohol consumption, and substances uses, were not considered in the comparison of general health status between men and women. Major sex differences, especially in terms of higher prevalence on smoking, may explain some of the poorer general health status in men.

In conclusion, based on thorough search and analysis of relevant literature, we provide a hypothesis in this paper that may explain the difference between the effect of cigarette smoking on long-term health outcomes of male and female smokers: pregnancy amplifies the effects of tobacco smoking, leading to higher risk of adverse long-term health outcomes in female smokers than in male smokers. This hypothesis could be tested by study designs proposed in this paper. Our hypothesis that pregnancy amplifies the consequence of cigarette smoking in female smokers, if proven true, may help in campaigns aiming at emphasizing the importance of quitting cigarette smoking during pregnancy and postpartum. Large scale, longitudinal studies in diverse populations with the design proposed by this paper would be particularly valuable.

## Abbreviations

CI, Confidence interval; CHD, Coronary Heart Disease; COPD, Chronic obstructive pulmonary disease; OR, Odds ratio; PaCO2, Partial Pressure of Carbon Dioxide; PaO2, Partial Pressure of Dioxide.
